# Antimicrobial Efficacy of a Taurolidine‐Based Antimicrobial Compound on Contaminated Surfaces Simulated in a Standardized 4‐Field Test

**DOI:** 10.1002/adhm.202503479

**Published:** 2025-11-12

**Authors:** Benito Baldauf, Hendrik Bonnemeier, Ernest W. Lau, Jana Hummel, Reinhard Vonthein, Ojan Assadian

**Affiliations:** ^1^ Institute of life science University of Applied Sciences Bremerhaven 27568 Bremerhaven Germany; ^2^ Medical Faculty Christian‐Albrechts University 24118 Kiel Germany; ^3^ Hospital Schildautal 38723 Seesen Germany; ^4^ Royal Victoria Hospital Belfast Belfast BT12 6BA UK; ^5^ IMBS University Lübeck 23562 Lübeck Germany; ^6^ Institute for Skin Integrity and Infection Prevention Human and Health Sciences University of Huddersfield Huddersfield HD1 3DH UK; ^7^ University Hospital Wiener Neustadt Wiener Neustadt 2700 Austria

**Keywords:** antimicrobial, cardiac implantable electronic device, implant surface decontamination, osteosynthesis material, taurolidine

## Abstract

Implantable medical devices, including cardiac electronic implants, joint prostheses, and breast implants, are essential to modern healthcare but remain susceptible to infection from microbial contamination during placement. *Staphylococcus spp*. and *Candida albicans* are the predominant pathogens, often causing severe complications, increased mortality, and substantial healthcare costs. With antibiotic resistance on the rise, intraoperative surface disinfection has emerged as a critical yet underutilized preventive strategy. Taurolidine, a broad‐spectrum antimicrobial with a strong safety profile and no known resistance, can be applied directly to both tissues and device surfaces. To replicate intraoperative decontamination, taurolidine‐saturated swabs are tested under standardized mechanical wiping using the European Norm EN 16615 “4‐field test.” The model reproduced short contact times and organic load conditions reflective of clinical practice, enabling assessment of both chemical and mechanical antimicrobial effects. Taurolidine achieved >5 log_10_ reductions in *Staphylococcus aureus*, *Pseudomonas aeruginosa*, and *Candida albicans*, and >4 log_10_ for *Enterococcus hirae*. The observed activity demonstrated a surface‐driven, enzyme‐independent mechanism involving spontaneous release of reactive N‐methylol functional groups that disrupt microbial membranes. Taurolidine's enzyme‐independent antimicrobial mechanism distinguishes it from solvent‐ or oxidant‐based disinfectants. These results support taurolidine as a potential adjunctive prophylactic agent to reduce device‐related infections during implantation procedures.

## Graphical abstract

As implantable medical devices become indispensable to modern medicine, a silent threat grows alongside them: device‐associated infections. Despite decades of antibiotic innovation, infection rates keep climbing, costing lives and billions in healthcare expenses. This study explores taurolidine, a safe, broad‐spectrum antimicrobial, as a potential breakthrough for direct device surface disinfection, targeting the problem where it truly begins.XXXX

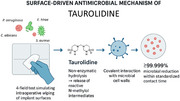

## 1 Introduction

The increasing reliance on implantable medical devices, from joint replacements to cardiac rhythm management, has led to a parallel rise in device‐associated infections. These infections contribute significantly to patient morbidity, mortality, and healthcare costs. For cardiac implantable electronic devices (CIEDs), which often require replacement due to battery depletion, infection risk is particularly high. Despite advances in prophylactic antibiotics and antiseptic protocols, infection rates have steadily increased over the last three decades.^[^ ^1^, ^2^, ^3^ ^]^ Conventional infection control strategies, such as systemic antibiotics and topical antiseptics, primarily target skin and circulating microbes, leaving the device surface relatively unprotected. Surface disinfection of the implants themselves is rarely practiced or standardized. This gap is clinically significant, especially considering the high mortality (8–17%) and cost (∼$51000 per case) associated with CIED infections.^[^ ^4^, ^5^, ^6^, ^7^, ^8^, ^9^, ^10^, ^11^ ^]^

In the majority of observed infections relating to implanted devices, *Staphylococcus* spp. are predominant,^[^ ^4^ ^]^ while fungal infections are less frequent, with *Candida albicans* being the most common fungal pathogen.^[^ ^12^ ^]^

Chemical agents used in infection prevention include antibiotics (systemic), antiseptics (for living tissue), and disinfectants (for inanimate surfaces). Some agents, such as povidone‐iodine (PVP‐I) and hydrogen peroxide, are used in both categories. Notably, intraoperative device irrigation with antibiotics lacks standardized guidelines or proven efficacy. Moreover, the overuse of systemic antibiotics has fueled antimicrobial resistance, limiting treatment options.^[^ ^13^, ^14^, ^15^, ^16^, ^17^ ^]^

Taurolidine is a broad‐spectrum antimicrobial with a longstanding record of safety. Unlike most disinfectants, it can be safely introduced into the bloodstream. Clinically, it has been used to irrigate surgical fields, flush catheters, and clean CIED hardware. Taurolidine's antimicrobial efficacy, lack of resistance, and tolerability make it a promising candidate for surface disinfection of implantable devices.^[^ ^18^, ^19^, ^20^, ^21^, ^22^, ^23^, ^24^, ^25^, ^26^, ^27^, ^28^, ^29^, ^30^, ^31^, ^32^, ^33^ ^]^

Despite promising clinical data, however, its mechanism of action when applied to device surfaces, particularly in the absence of esterase‐driven hydrolysis, remains poorly understood. Prior studies have relied on long immersion times, which do not mimic real‐world clinical settings. To our knowledge, no prior study has quantitatively assessed taurolidine's antimicrobial performance when applied via mechanical wiping under standardized conditions simulating surgical device handling. Understanding this behavior is essential to bridge the gap between taurolidine's systemic antiseptic use and its potential as a surface‐active antimicrobial for medical materials, such as CIEDs or osteosynthesis materials. The testing followed European Norm EN 16 615, a validated method (the “4‐field test”) for assessing disinfectant wipe effectiveness. ^[^ ^29^, ^31^, ^34^, ^35^, ^36^, ^37^, ^38^ ^]^

## 2 Experimental Section

### 2.1 Preparation: Microbial suspensions

Reference microbial strains from the American Type Culture Collection (ATCC), including *Staphylococcus aureus* (ATCC 6538), *Pseudomonas aeruginosa* (ATCC 15 442), *Enterococcus hirae* (ATCC 10 541), and *Candida albicans* (ATCC 10 231), were grown on tryptic soy agar (TSA, Oxoid, catalog: CM0131R) and incubated at 36 °C ± 1.0 °C for 48 h (Binder BDS115‐230 V). Initial subcultures were prepared using sterile 1 µL inoculating loops (Roth selection), diluted in 1 mL of NaCl peptone (NaP, Oxoid, catalog: CM0982B), plated on TSA, and incubated under the same conditions.

**Figure 1:**
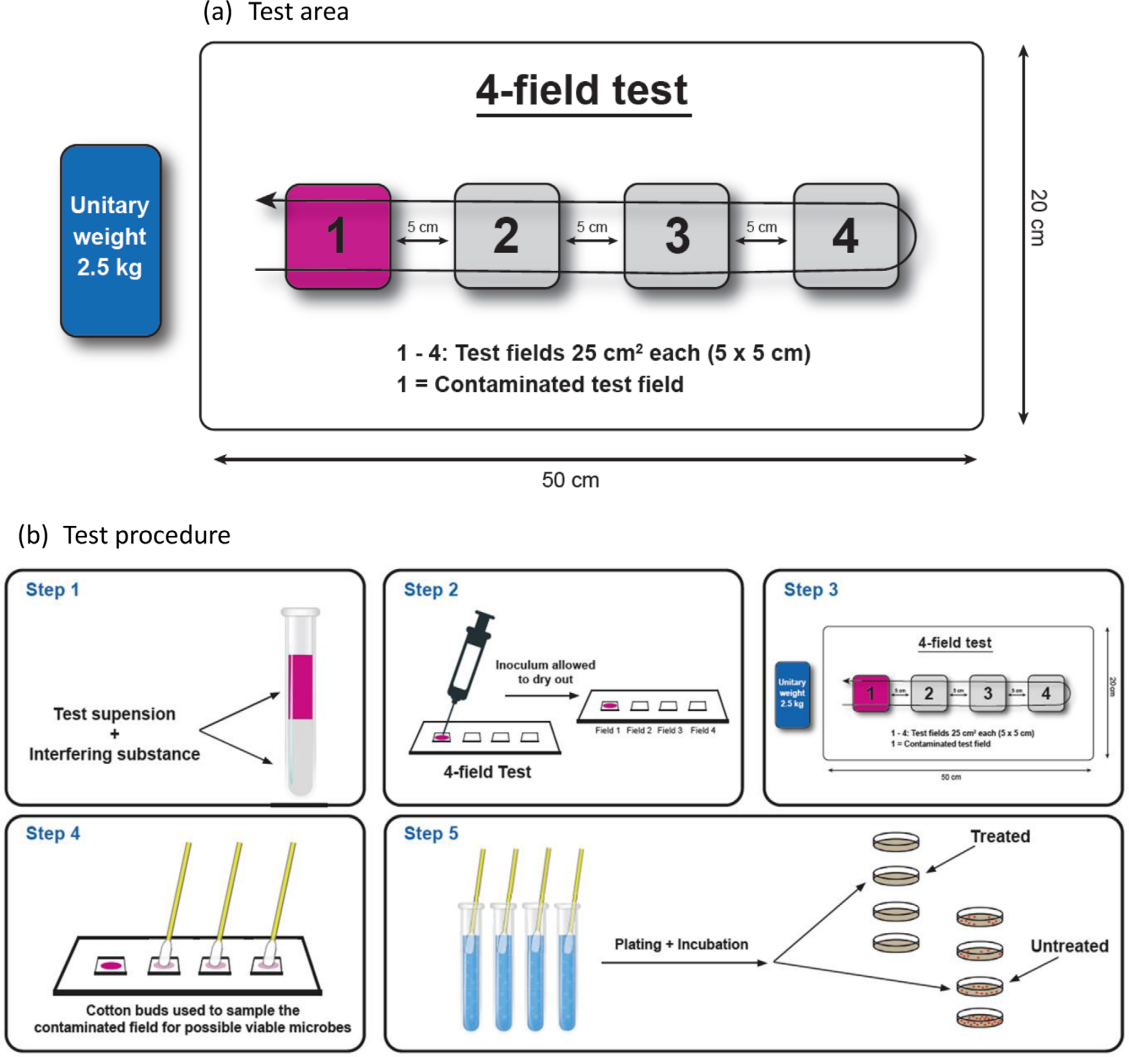
The 4‐field test a) Test area: The test setup consists of four adjacent 5 × 5 cm fields (25 cm² each) arranged within a 50 × 20 cm pvc plate. Field 1 is the contaminated test area, while fields 2–4 represent uncontaminated adjacent areas. A 2.5 kg unitary weight is moved across the fields to simulate surface transfer during cleaning or disinfection. b) Test procedure: Step 1: Preparation of the test suspension. Step 2: The inoculum is applied to Field 1 (contaminated area) and allowed to dry. Step 3: A unitary weight (2.5 kg) is moved once across the four test fields to simulate wiping and potential microbial transfer. Step 4: Cotton swabs are used to sample each field for the presence of viable microorganisms. Step 5: Samples are transferred to tubes containing neutralizer, plated on agar, and incubated to determine microbial recovery from treated and untreated fields.

Subsequent subcultures were obtained with sterile inoculating loops, diluted in 1 mL of NaP, and combined with 0.03% bovine serum albumin (BSA, Merck, catalog: 1 120 180 100). The BSA was included to simulate an organic challenge that could interfere with disinfectant performance under clinical conditions (**Figure** **1**). The microbial count of the test suspension was adjusted to a density of 1.5–5.0 × 10^9^ CFU mL^−1^ for bacteria and 1.5–5.0 × 10^8^ CFU mL^−1^ for *C. albicans*, estimated by DEN‐1B McFarland Densitometer (Grant Instruments Ltd., England).

### 2.2 Justification of Inoculum Density

Test organism suspensions were prepared to the inoculum densities required by EN 16 615 (bacteria: 1.5–5.0 × 10^9^ CFU mL^−1^; *Candida albicans*: 1.5–5.0 × 10^8^ CFU mL^−1^). These high inocula are deliberately used by the EN 16 615 “4‐field” method to create a worst‐case contamination challenge that simultaneously evaluates 1) the chemical bactericidal/yeasticidal activity and 2) the mechanical removal and potential spread of viable microbes during wiping. The standard's pass/fail criteria (≥5 log_10_ reduction on the inoculated field and ≤50 CFU mean on fields 2–4) are calibrated to those high starting counts and therefore require high starting inocula to remain interpretable. Using this standardized, conservative challenge allows direct comparison with other disinfectant/wipe evaluations and ensures that observed activity is robust under demanding conditions. This test design not only enables comparison with standardized disinfectants but also provides a reproducible model for studying the physical and chemical interaction between taurolidine and microbial contaminants under dynamic wiping forces. Conditions previously unexplored in taurolidine research.

### 2.3 Preparation: Test Area and Fields

Homogeneous polyvinyl chloride (PVC) sheets measuring 50 × 20 cm were used to mimic the non‐porous surface of implanted medical devices and prostheses. Each sheet was divided into four separate 5 × 5 cm test fields using a marker, with 5 cm spacing between the fields (Figure 1a). The PVC test sheets were immersed in 100 mL of 70% isopropyl alcohol for 15 min, dried under laminar airflow, and stored in a cleanroom environment classified as Class 5 (ISO EN 14 644) to prevent contamination before use.

### 2.4 Preparation: Test Liquid Swabs

Low‐dusting non‐woven fabric swabs 17 × 30 cm in size (TORK, Essity Professional Hygiene Germany GmbH) were saturated with 16 mL of the test solution, either taurolidine as the disinfectant or sterile water as the control, and were used to apply the test solutions to the test fields. Taurolidine‐saturated swabs were prepared immediately before each test run and employed without storage (commercially available in vials only). Consequently, pH and chemical stability during storage were not assessed, as storage of test swabs is not part of the standardized method.

### 2.5 Preparation: Neutralizer Solution

A validated solution containing Tween 80 (polysorbate, Carl Roth GmbH + Co. KG, Karlsruhe, Germany) at 30 g L^−1^, saponin (Carl Roth) at 30 g L^−1^, L‐histidine (Carl Roth) at 1 g L^−1^ and cysteine (Carl Roth) at 1 g L^−1^ was used as neutralizer to inactivate the antimicrobial activity of the test disinfectant.

### 2.6 Simulated Microbial Contamination of the Test Field

The test procedure was conducted at a controlled temperature of 22.5°± 2.5 °C. In step 1 (Figure 1b), 50 µL of each microbial test solution was drawn. Subsequently (step 2), this suspension was applied to the center of field 1 within the test area using a sterile glass spatula (Karl Hammacher GmbH, Solingen, Germany). The test area was then allowed to dry under clean room conditions, class 5 following ISO EN 14 644 for 60 min to prevent cross‐contamination and to facilitate the attachment of test strains to the test surface. All experiments were conducted in triplicate following the ISO EN 16 615.

### 2.7 Simulated Antimicrobial Treatment of Contaminated Surface

A unitary weight, represented by a 2.5 kg steel‐block covered with a test liquid swab, was manually wiped across the test area. The wiping motion proceeded from field 1 to field 4 and then back to field 1. The dwell time for each test field was kept under 1s, as indicated in step 3 (Figure 1b). The contact time between the test liquid and the standardized microbial contamination retrieval was set at 5 min, starting when the wipe concluded and the weight returned to field 1. The swab's weigh was measured gravimetrically immediately before and after use to assess indirectly the amount of the applied test solution on the test surface.

### 2.8 Testing the Test Fields for Viable Microbes After Contact with Disinfectant

Following contact with the test solution, each test field underwent a two‐step swabbing process. Initially, a wet swab saturated with the neutralizer was used, followed by a dry swab (step 4, Figure 1b). The portion of the wet and dry swabs that came into direct contact with the test field's surface was rinsed in a tube containing neutralizer to produce a test‐neutralizer mixture (TNM). After allowing 10 s for the neutralizer to take effect, a 1 mL sample was extracted from the TNM and subjected to a tenfold dilution (step 5, Figure 1b). Subsequently, two 1 mL samples of the diluted TNM were plated on TSA and incubated to facilitate microbial growth.

### 2.9 Drying Control D_C0_ and D_Ct_

In order to quantify the viability of the test microbes from drying alone over time without any mechanical and chemical influences from wiping with a disinfectant, two control‐test fields, D_C0_ and D_Ct,_ identical to field 1 in dimensions but physically separate from the 4‐field test area, were contaminated in the same fashion.^[^ ^39^ ^]^ Sampling of test field D_C0_ occurred immediately after drying and before wiping the contaminated test fields (i.e., contact time 0, or C0), whereas sampling of test field D_Ct_ occurred after the contact time between the test microbes and the test disinfectant had elapsed (i.e., contact time *t*, or Ct).

### 2.10 Testing the Neutralizer for Ability to Inactivate Disinfectants (Control A)

A 9 mL disinfectant‐neutralizer mixture, comprising 0.2 mL of the test disinfectant and 8.8 mL of the neutralizer, was incubated at 20 °C for 10 s in a water bath. This mixture was then added to 1 mL of the microbial suspension. After incubation at 20 °C for 30 min in a water bath, two 1 mL samples (x and x’) were extracted from the disinfectant‐neutralizer‐microbe mixture and plated on TSA to observe microbial growth.

### 2.11 Testing the Neutralizer for Microbicidal Activity (Control B)

A 10 mL mixture consisting of 8 mL of the neutralizer, 1 mL of sterile water, and 1 mL of microbial suspension was incubated at 20 °C for 5 min. Two 1 mL samples (x and x’) were taken from the mixture and plated on TSA to assess microbial growth.

### 2.12 Statistical Analysis

Statistical analysis was performed following the EN 16 615 standard, the four‐field test for evaluating the efficacy of disinfectant wipes. Colony‐forming units (CFU) were enumerated from the initial test suspension (N), drying controls before and after contact time (DC0, DCt), water control fields (Nw), test fields (Na), and disinfectant‐treated fields (VT2–4). Logarithmic transformations of CFU counts were calculated, and reduction factors (R) were determined by comparing the test suspension to disinfectant‐treated fields. Descriptive statistics, including ranges and means, were used to assess reproducibility, and the amount of liquid released from the wipes was measured to ensure consistent application. Statistical analysis of the EN 16 615 four‐field test was performed using R (version 3.4).

## 3 Result

**Figure 2:**
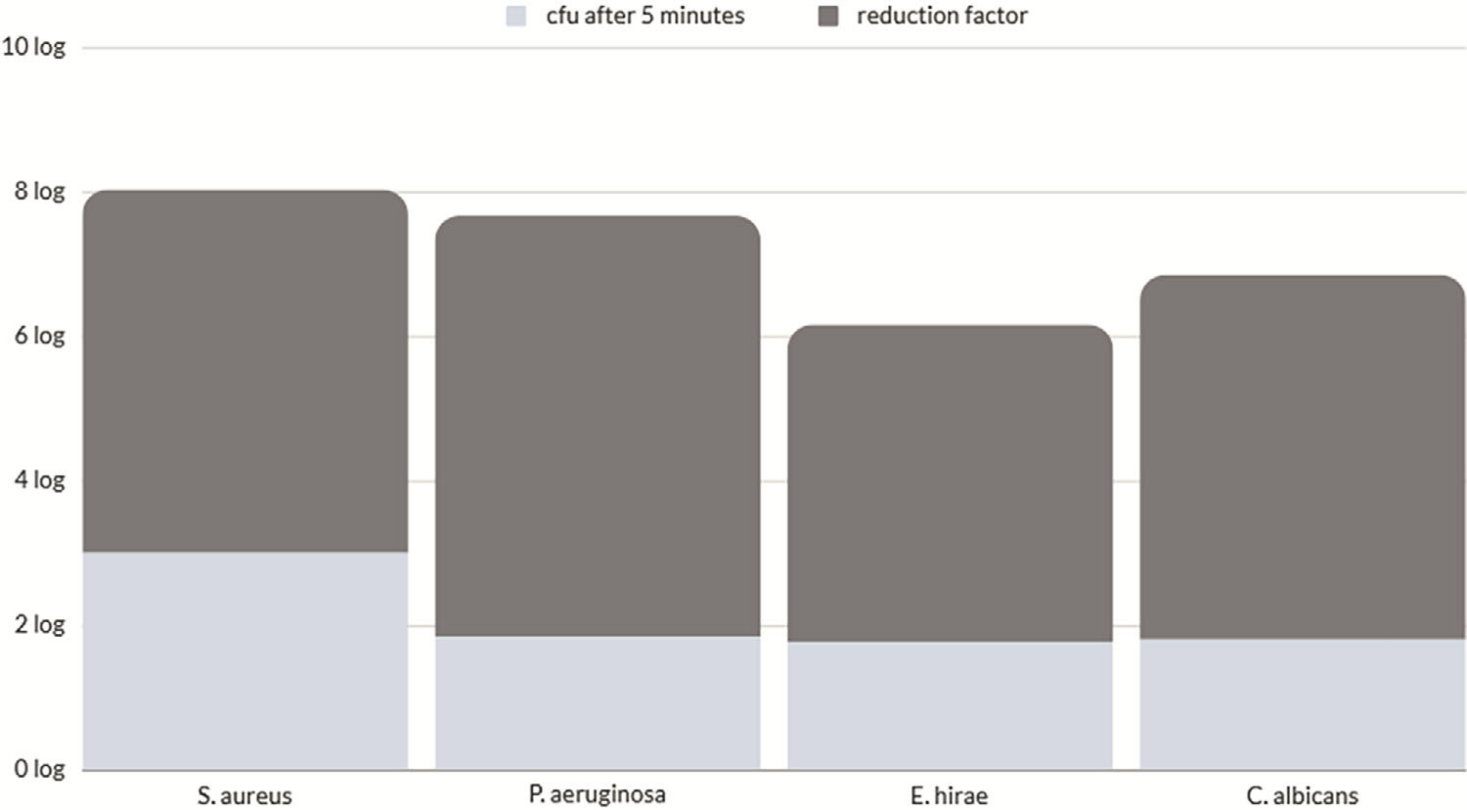
Diagram showing the log_10_ reduction of the microbial strains tested.

Following a standardized testing methodology for the antimicrobial efficacy of test solutions on inanimate surfaces, taurolidine exhibited significant efficacy as a surface disinfectant, achieving a microbial reduction of >5 log_10_ for Staphylococcus aureus, Pseudomonas aeruginosa, and Candida albicans, and > 4 log_10_ for Enterococcus hirae. The detailed results of the experiment are summarized in **Table** **1** and **Figure** **2**, and Table S1 (Supporting Information), respectively. The magnitude of reduction achieved under brief, mechanically assisted contact supports the hypothesis that taurolidine exerts rapid surface‐level antimicrobial action independent of enzymatic hydrolysis. This finding provides experimental evidence of its reactivity in a nonbiological environment, previously hypothesized but not quantitatively demonstrated.

**Table 1:** Log_10_ reduction in the number of colony‐forming units (CFU) of microbial strains after exposure to Taurolidine in the 4‐field test.

Test strain (CFU count per mL)	Log_10_ reduction	Met EN 16615	Met USP‐NF 1072
*Staphylococcus aureus* (10^9^ CFU mL^−1^)	= 5.03	✓	✓
Enterococcus hirae (10^7^ CFU mL^−1^)	>4.39		✓
*Pseudomonas aeruginosa* (10^9^ CFU mL^−1^)	> 5.83	✓	✓
*Candida albicans* (10^8^ CFU/mL)	> 5.05	✓	✓

EN, European Norm; USP‐NF, United States Pharmacopeia–National Formulary

## 4 Discussion

This study provides the first systematic evaluation of taurolidine's antimicrobial performance under dynamic wiping conditions that simulate intraoperative handling of implantable medical devices. Unlike previous investigations that focused on its systemic or catheter‐based applications, our findings demonstrate that taurolidine retains potent, rapid antimicrobial activity even in the absence of enzymatic hydrolysis. This observation suggests a non‐enzymatic, surface‐mediated mechanism of action, in which spontaneous release of reactive N‐methylol functional groups contributes to microbial membrane disruption. The results therefore, extend taurolidine's known spectrum of activity from liquid or intraluminal contexts to direct application on non‐porous device surfaces, a translational step that had not been experimentally quantified before.

Although the standardized inoculum used in the EN 16 615 “four‐field test” exceeds typical clinical contamination levels, such a stringent challenge models the real‐world difficulty of eradicating sessile bacterial communities found in CIED pockets and other biofilm‐prone surgical environments. This conservative testing approach ensures that the demonstrated reductions reflect robust efficacy under demanding conditions and provide meaningful insight into taurolidine's potential for preimplant surface disinfection.

The solution researched is certified for adjunctive use during CIED and prosthetic joint replacement procedures. It is employed as an antiseptic irrigation in the surgical site and as a disinfectant mechanically applied to the CIED components (e.g., indwelling and de novo leads or generator via soaked swabs) and osteosynthesis materials. During the test, simulating the treatment of any implantable medical device, the Taurolidine‐containing solution was able to hugely reduce the microbial count on a standardized surface.

### 4.1 Generation and Biological Actions of the Metabolites of Taurolidine

When taurolidine is dissolved in an aqueous povidone solution, it is only minimally hydrolyzed to N‐methylol compounds. Upon initiation of taurolidine use (i.e., in the human body), taurolidine undergoes further hydrolysis mediated by esterases and other enzymes, resulting in the formation of active metabolites, namely N‐methylol taurultam, taurultam, and N‐methylol taurinamide. These metabolites exhibit half‐lives from 1 to 6 h in vitro, particularly in the presence of saccharides, peptides, and glycans.^[^ ^24^ ^]^ Each taurolidine molecule yields three N‐methylol groups, believed to be the chemically active species responsible for taurolidine's antimicrobial effect.^[^ ^23^, ^24^, ^25^, ^26^, ^27^, ^28^ ^]^ These effects encompass the denaturation of the pathogen's cell wall, with or without peptidoglycans, fimbriae, or flagellates.^[^ ^40^ ^]^ Additionally, taurolidine demonstrates inhibitory actions on the attachment to epithelial, osteoblast, and fibroblast cells, disruption of biofilm formation, and neutralization of endotoxins and certain exotoxins.^[^ ^23^, ^41^ ^]^

Further metabolic processes transform the active metabolites into taurine, an amino‐sulfonic acid naturally occurring in biological tissues and secretions. Taurine is recognized for its antioxidant and anti‐inflammatory properties, and studies have indicated its ability to enhance wound healing in both in vitro and in vivo animal models.^[^ ^19^, ^42^, ^43^, ^44^, ^45^, ^46^ ^]^ Notably, taurine also contributes to the creation of an acidic microenvironment, promoting the hydrolysis of taurolidine and the generation of active metabolites.

Over some time, concerns existed that formaldehyde may be formed within taurolidine solutions. The potential presence of formaldehyde would be of significance because if released, it could damage eukaryotic cells by inducing DNA damage, promoting inflammatory responses, and causing cytotoxic effects. Yet, taurolidine is a synthetic derivative of taurine and is recognized for its broad‐spectrum antimicrobial properties. Under physiological conditions, taurolidine undergoes hydrolytic reactions that generate several active metabolites. These include taurultam derivatives and reactive N‐methylol groups, which are believed to be responsible for the antimicrobial action through mechanisms such as the inactivation of bacterial endotoxins and the inhibition of cell adhesion. Importantly, although these reactive intermediates are chemically active, it has been demonstrated that they do not liberate free formaldehyde during their conversion. Instead, the N‐methylol groups remain covalently bound or are rapidly transformed into non‐toxic compounds. Indeed, Knight et al.^[^ ^47^ ^]^ employed advanced analytical techniques, including nuclear magnetic resonance and gas chromatography analysis, to investigate the metabolic fate of taurolidine. Their analyses demonstrated that taurolidine's conversion under physiological conditions does not produce measurable amounts of free formaldehyde. Consequently, the absence of formaldehyde release helps to explain taurolidine's favorable safety profile when used as an antimicrobial agent.

### 4.2 Microbial Efficacy of Taurolidine as Antiseptic/Disinfectant

In the 4‐field test, taurolidine achieved a ≥5 log_10_ reduction for *S. aureus*, *P. aeruginosa*, and *C. albicans*, and a ≥4 log_10_ reduction for *E. hirae*, meeting or closely approaching the requirements of EN 16 615 and USP–NF standards. Beyond confirming its broad‐spectrum antimicrobial performance, these results reveal an important mechanistic insight: the observed activity indicates that taurolidine's antimicrobial mechanism remains functional even in the absence of esterase‐mediated hydrolysis. This supports a surface‐driven reaction mechanism, likely involving the spontaneous release of reactive *N‐methylol* intermediates upon contact with organic residues on contaminated surfaces. These intermediates can interact covalently with microbial envelope structures, leading to rapid destabilization of membranes and cellular components.^[^ ^25^ ^]^

Such behavior distinguishes taurolidine from conventional disinfectants, which rely primarily on solvent (ethanol), oxidative (hydrogen peroxide, hypochlorous acid), or membrane‐disruptive (chlorhexidine, PHMB) pathways. Instead, taurolidine appears to operate through a controlled chemical reaction mechanism that remains active without enzymatic catalysis, explaining its efficacy under short contact times and non‐biological conditions. This surface‐reactive property highlights taurolidine's unique potential as an antimicrobial coating or intraoperative surface treatment, bridging the gap between classical antiseptics and functional antimicrobial materials.

### 4.3 Deep vs Superficial Wounds; Closed Versus Open Cavities

Furthermore, the versatility of taurolidine is evident in its applicability to both deep wounds and closed cavities, in contrast to many established antiseptics limited to use in superficial wounds exposed to the external environment (PHMB, PVP‐I, OCT, H_2_O_2_, ethanol). Taurolidine can be safely employed in deep wounds or closed cavities, such as CIED pockets, prosthetic joint cavities, and deep incisional wounds.^[^ ^18^, ^48^, ^49^ ^]^

### 4.4 Interference with Surface Attachment and Biofilm Formation

Bacteria exist in two primary states: planktonic and biofilm‐associated. Planktonic bacteria are single cells. In contrast, biofilm‐associated bacteria form complex, multicellular communities attached to surfaces, encased in a self‐produced extracellular matrix, the biofilm. This matrix provides structural support and facilitates intercellular communication (i.e.,; quorum sensing). The transition from planktonic to biofilm state is influenced by environmental factors and genetic regulation. Biofilm formation enhances bacterial survival by providing protection against environmental stresses, including nutrient deprivation, pH changes, and exposure to antibiotics. For instance, bacteria within biofilms exhibit increased resistance to antimicrobial agents compared to their planktonic counterparts. Additionally, biofilm‐associated bacteria can exchange genetic information more readily, contributing to the spread of antibiotic resistance. Understanding the dynamics between these two states is crucial for developing effective strategies to prevent and treat bacterial infections. In terms of interference with surface attachment and biofilm formation, exposure of microbes to sublethal concentrations of common antiseptics may promote the formation of biofilm, a highly effective defense against chemical agents.^[^ ^42^, ^43^, ^44^ ^]^ Taurolidine, on the other hand, disrupts the attachment of microbes to surfaces,^[^ ^34^, ^40^ ^]^ and inhibits progressing colonization and biofilm formation, and hence, may prevent primary infection.^[^ ^41^, ^50^ ^]^

### 4.5 Medium Half‐Lives of Taurolidine and its Metabolites

Antibiotics disrupt energy‐dependent processes in bacterial cells, making disturbances to metabolic homeostasis a critical aspect of their action. The relationship between bacterial metabolism and antibiotic efficacy can be understood through three interconnected principles. Antibiotic treatment alters the metabolic state of bacteria, contributing to cell death (bacteriocidic) or growth arrest (bacteriostatic). Conversely, the pre‐existing metabolic state of bacteria significantly influences their susceptibility to antibiotics, shaping the outcome of treatment. Furthermore, the effectiveness of antibiotics can be enhanced by deliberately modulating bacterial metabolism. Thus, attempting to irrigate the surgical site or wipe a medical prosthesis with diluted antibiotics designed for repeat intravenous use over an extended period is unlikely to attain a local drug concentration sufficient to produce significant bactericidal or bacteriostatic effects. The sustained maintenance of antimicrobial actions with antibiotics typically necessitates their gradual release from an implanted reservoir within the body.^[^ ^51^, ^52^ ^]^ In contrast, taurolidine and its active metabolites can achieve sustained antimicrobial actions lasting over 24 h. This prolonged action is facilitated by the progressive hydrolysis of taurolidine, resulting in active metabolites with half‐lives ranging from 1 to >6 h.^[^ ^24^ ^]^ Notably, this sustained antimicrobial activity can be achieved through a straightforward process, such as the instillation of taurolidine into the surgical site and even more so by wiping the surface of the medical prosthesis.

### 4.6 Clinical Implications

Two recently published studies,^[^ ^18^, ^53^ ^]^ encompassing over 2200 CIED procedures, observed that the use of taurolidine resulted in a remarkable reduction of the acute infection rate to 0.0% and 0.125% respectively. In these studies, taurolidine‐soaked swabs were employed to wipe CIED components before placement and to irrigate the surgical site after CIED placement, including the fibrous CIED pocket for generator substitution, upgrade, or downgrade revision, extraction, or the preparation of a new pocket for de novo CIED placement.

The precise mechanisms underlying the observed antimicrobial efficacy of taurolidine are not fully understood. In the 4‐field test, swabs were fully saturated with taurolidine immediately prior to wiping, as per the standardized protocol. Taurolidine exerts its antimicrobial activity primarily through the release of N‐methylol groups, which disrupt microbial cell envelopes. Although the solution is typically pH‐neutral, the intrinsic chemical properties of taurolidine, including its reactivity and N‐methylol release, likely contribute to the effective reduction of bacterial and fungal colony‐forming units observed during the test. This activity occurs independently of any unmeasured changes in pH during swab preparation or use. Importantly, the commercially available formulation of taurolidine used in this study does not contain ethanol or quaternary ammonium compounds, indicating that the antimicrobial effects are attributable to taurolidine itself rather than auxiliary ingredients.

Therefore, the test conducted and elucidated in this manuscript provides valuable insights into the properties of Taurolidine that were previously a subject of suspicion or hypothesis. These findings contribute to enhancing clinical practices during the placement of CIEDs or osteosynthesis materials with the adjunctive use of taurolidine (i.e., mechanical application via soaked swabs to indwelling hardware to potentially disrupt biofilms and/or prevent attachment of planktonic pathogens^[^ ^50^, ^54^ ^]^).

The clinical relevance of the results could be further enhanced by conducting the same test using microorganisms isolated from actual clinical infections.

### 4.7 Methodological Considerations

The EN 16 615 “four‐field test” was selected to evaluate the efficacy of a commercially available taurolidine‐containing solution on a standardized non‐porous surface representative of medical‐device materials. Although microbial loads in operating rooms or CIED generator pockets are typically lower than the inocula specified by this standard, device pockets and biofilm‐forming sites can harbor locally high bacterial densities and sessile communities that are more resistant to removal than planktonic cells. Using a conservative, high‐level challenge, therefore, provides a stringent model that approximates the practical difficulty of disinfecting medical device surfaces in clinical settings.

### 4.8 Limitations

Several limitations should be considered when interpreting our findings. First, the wiping procedure employed in this study used a standardized 2.5 kg block, as prescribed by EN 16 615, to ensure reproducibility and comparability of results. While this approach is innovative, it does not fully reflect the variability and nuance of manual swab handling during device implantation or revision surgery. Therefore, the present results should be viewed as a conservative approximation of clinical practice rather than a direct replication.

Second, the study was conducted using a standardized wipe‐test designed primarily for evaluating surface disinfectants, such as those commercially manufactured for use on countertops, diagnostic equipment, or operating theatre stretchers. In this assay, polyvinyl chloride (PVC) serves as a surrogate material. Whether this adequately simulates the physicochemical properties of CIED components or osteosynthesis materials made of stainless steel, titanium, iridium, ceramics, epoxy resin, or silicone remains uncertain. Nevertheless, the standardized wipe test was deemed a feasible preliminary step in the absence of contemporary data on the preclinical activity of taurolidine.

Third, the standardized wipe test includes only four reference strains. While *Staphylococcus* spp. and *Candida albicans* are highly relevant to CIED infections,^[^ ^4^ ^]^ other potential pathogens were not represented. Notably, *Enterococcus hirae*, included as a representative of enterococci, is rarely implicated in clinical device infections. Taurolidine narrowly failed to meet European requirements for *E. hirae* (>5 log_10_ reduction) but did achieve the USP‐NF criterion (>4 log_10_ reduction). Given that no clinical cases of *E. hirae* CIED infection have been reported, and that other enterococci (*E. faecalis*, *E. cloacae*) are only rare causes of CIED infection, the clinical significance of this finding is uncertain. During device placement, a microbial reduction exceeding 4 log_10_ is generally considered sufficient to prevent infection development.

Finally, while metabolic flexibility in *E. hirae* could contribute to its relative resilience, taurolidine's antimicrobial mechanism, disruption of microbial cell envelopes via release of N‐methylol groups, is not dependent on specific metabolic pathways. Its broad‐spectrum efficacy, including activity against multiple *Enterococcus* species, suggests that observed differences in *E. hirae* responses may be attributable to structural features of the bacterial envelope rather than unique metabolic adaptations. Further studies using clinically derived CIED isolates and different surface materials are warranted to confirm and extend these findings.

Nevertheless, this standardized model provides a necessary mechanistic bridge between taurolidine's biochemical reactivity and its practical antimicrobial function on implantable materials, warranting further surface‐chemistry and biofilm studies.

## Conflict of Interest

Benito Baldauf reports relationships with Abbott, Kimal plc, Kappamed, Cablon NL, Crosstec, Bioline Supply, Biotronik, MCM Ag, Medival SRL, Medztronic, Philips/Spectranetics, Tauro‐Implant, TauroPharm GmbH, Transcutan, Stereotaxis, and 4G involving board membership, consulting/advisory roles, paid expert testimony, speaking/lecture fees, and travel reimbursement. He also reports that article publishing charges are covered by the University of Applied Sciences Bremerhaven. Hendrik Bonnemeier and Jana Hummel report that article publishing charges are covered by the University of Applied Sciences Bremerhaven. Reinhard Vonthein reports a research grant provided to his employer by TauroPharm GmbH related to a different manuscript. Ernest W. Lau and Ojan Assadian report no conflicts of interest.

## Author Contributions

R.V. and O.A. contributed equally to this work. B.B. performed data curation, methodology, supervision, and wrote the original draft preparation. H.B.performed conceptualization, methodology, wrote, reviewed, and edited the final draft, and supervision. E.W.L. wrote, reviewed, and edited the final draft. J.H. and O.A. wrote, reviewed, and edited the final draft, supervision. R.V. performed conceptualization and validation.

## Supporting information



Supporting Information

## Data Availability

The data that support the findings of this study are available in the supplementary material of this article.
