# Supplementary material for: Antimicrobial Efficacy of a Taurolidine‐Based Antimicrobial Compound on Contaminated Surfaces Simulated in a Standardized 4‐Field Test
Source: Adv Healthc Mater. 2025 Nov 12;15(7):e03479. doi: 10.1002/adhm.202503479 (PMC12908203; doi:10.1002/adhm.202503479)
Supplement: Supplementary file 1 — Supporting Information [file ADHM-15-0-s001.docx]

Table S1. Detailed results of the 4-field test for a specific Taurolidine 2% solution commercially available and different microbial strains

| N | D_C0_ | D_Ct_ | N_w_ | N_a_ | V_T2-4_ |
| --- | --- | --- | --- | --- | --- |
| Staphylococcus aureus (strain ATCC 6538) | | | | | |
| 10^-7^: 202 \| 140  10^-8^: 19 \| 19 | 10^-5^: 96 \| 135  10^-6^: 15 \| 15 | 10^-5^: 229 \| 209  10^-6^: 24 \| 20 | 2: 10^0^: 4 \| 3  2: 10^-1^: 0 \| 0  3: 10^-0^: 3 \| 3  3: 10^-1^: 0 \| 0  4: 10^-0^: 1 \| 1  4: 10^-1^: 0 \| 0 | 10^0^: 204 \| 204  10^-1^: 21 \| 26  10^-2^: 2 \| 2  1.030 CFU/mL | 2: 9.5 (10 \| 9)  3: 0 (0 \| 0)  4: 0 (0 \| 0) |
| **9.24 log** | **7.77 log** | **8.04 log** | **12.5 CFU** | **3.01 log** | **15.83 CFU** |
| 9.17 < N < 9.70 | 6.88 < D_C0_ < 8.40 | 6.88 < D_Ct_ < 8.40 | > 10 CFU | **R= 5.03** | < 50 CFU |
| Liquid released from wipe (grams) | | Test: **0.51** | | Control: **0.62** | |
| **Pseudomonas aeruginosa (strain ATCC 15442)** | | | | | |
| 10^-7^: 245 \| 255  10^-8^: 23 \| 23 | 10^-5^: 90 \| 89  10^-6^: 7 \| 13 | 10^-5^: 98 \| 93  10^-6^: 6 \| 10 | 2: 10^0^: 10 \| 12  2: 10^-1^: 2 \| 1  3: 10^-0^: 7 \| 4  3: 10^-1^: 0 \| 0  4: 10^-0^: 7 \| 5  4: 10^-1^: 2 \| 0 | 10^0^: 10 \| 6  10^-1^: 0 \| 0  10^-2^: 0 \| 0  < 70 CFU/mL | 2: 5.0 (6 \| 4)  3: 2.5 (3 \| 2)  4: 3.5 (5 \| 2) |
| **9.39 log** | **7.65 log** | **7.68 log** | **39.22 CFU** | **<1.85 log** | **18.33 CFU** |
| 9.17 < N < 9.70 | 6.88 < D_C0_ < 8.40 | 6.88 < D_Ct_ < 8.40 | > 10 CFU | **R > 5.83 log** | < 50 CFU |
| Liquid released from wipe (grams) | | Test: **0.57** | | Control: **0.49** | |
| **Enterococcus hirae (strain ATCC 10541)** | | | | | |
| 10^-5^: 186 \| 180  10^-6^: 29 \| 25 | 10^-3^: >330 \| >330  10^-4^: 38 \| 40 | 10^-3^: 98 \| 93  10^-4^: 34 \| 36 | 2: 10^0^: 3 \| 3  2: 10^-1^: 0 \| 0  3: 10^-0^: 8 \| 5  3: 10^-1^: 0 \| 0  4: 10^-0^: 3 \| 1  4: 10^-1^: 0 \| 0 | 10^0^: 8 \| 6  10^-1^: 0 \| 0  10^-2^: 0 \| 0  < 70 CFU/mL | 2: 1.5 (2 \| 1)  3: 1.0 (2 \| 0)  4: 1.0 (2 \| 0) |
| **7.28 log** | **6.29 log** | **6.24 log** | **19.17 CFU** | **<1.85 log** | **5.83 CFU** |
| 7.17 < N < 7.70 | 4.88 < D_C0_ < 6.40 | 4.88 < D_Ct_ < 6.40 | > 10 CFU | **R > 4.39 log** | < 50 CFU |
| Liquid released from wipe (grams) | | Test: **0.66** | | Control: **0.74** | |
| **Candida albicans (strain ATCC 10231)** | | | | | |
| 10^-6^: 238 \| 276  10^-7^: 26 \| 26 | 10^-4^: 205 \| 184  10^-5^: 22 \| 14 | 10^-4^: 152 \| 162  10^-5^: 19 \| 17 | 2: 10^0^: 2 \| 2  2: 10^-1^: 0 \| 0  3: 10^-0^: 3 \| 2  3: 10^-1^: 0 \| 0  4: 10^-0^: 3 \| 3  4: 10^-1^: 0 \| 0 | 10^0^: 13 \| 10  10^-1^: 2 \| 1  10^-2^: 0 \| 0  < 70 CFU/mL | 2: 4.5 (6 \| 4)  3: 2.0 (3 \| 2)  4: 6.0 (5 \| 2) |
| **8.41 log** | **6.98 log** | **6.90 log** | **12.50 CFU** | **<1.85 log** | **20.83 CFU** |
| 8.17 < N < 8.70 | 5.88 < D_C0_ < 7.40 | 5.88 < D_Ct_ < 7.40 | > 10 CFU | **R > 5.05 log** | < 50 CFU |
| Liquid released from wipe (grams) | | Test: **0.67** | | Control: **0.67** | |
| Result **in bold letters**  CFU colony forming units  N common logarithm of CFU (test suspension)  N_a_ test field 1; CFU count per test field  N_w_ water control test fields 2 – 4; CFU count per test field  V_T2-4_ disinfectant test fields 2 – 4; CFU count per test field  D_C0_ common logarithm of CFU (drying control)  D_Ct_ common logarithm of CFU (drying control after contact time)  R the factor of reduction | | | | | |
